# Brain Dynamics of Speech Modes Encoding: Loud and Whispered Speech Versus Standard Speech

**DOI:** 10.1007/s10548-025-01108-z

**Published:** 2025-02-15

**Authors:** Bryan Sanders, Monica Lancheros, Marion Bourqui, Marina Laganaro

**Affiliations:** Faculty of Psychology and Educational Sciences, University of Geneva, 40 Boulevard du Pont d’Arve, 1205 Geneva, Switzerland

**Keywords:** Amplitudes, Electroencephalography (EEG), Event related potentials (ERPs), Microstates, Motor speech control

## Abstract

Loud speech and whispered speech are two distinct speech modes that are part of daily verbal exchanges, but that involve a different employment of the speech apparatus. However, a clear account of whether and when the motor speech (or phonetic) encoding of these speech modes differs from standard speech has not been provided yet. Here, we addressed this question using Electroencephalography (EEG)/Event related potential (ERP) approaches during a delayed production task to contrast the production of speech sequences (pseudowords) when speaking normally or under a specific speech mode: loud speech in experiment 1 and whispered speech in experiment 2. Behavioral results demonstrated that non-standard speech modes entail a behavioral encoding cost in terms of production latency. Standard speech and speech modes’ ERPs were characterized by the same sequence of microstate maps, suggesting that the same brain processes are involved to produce speech under a specific speech mode. Only loud speech entailed electrophysiological modulations relative to standard speech in terms of waveform amplitudes but also temporal distribution and strength of neural recruitment of the same sequence of microstates during a large time window (from approximatively − 220 ms to − 100 ms) preceding the vocal onset. Alternatively, the electrophysiological activity of whispered speech was similar in nature to standard speech. On the whole, speech modes and standard speech seem to be encoded through the same brain processes but the degree of adjustments required seem to vary subsequently across speech modes.

## Introduction

Speech production is a complex cognitive-motor ability which allows humans to transform an abstract linguistic code into precise motor commands needed to produce an utterance. However, multiple intrinsic and extrinsic factors can interfere with the transmission of the message between a speaker and a listener. Therefore, speakers will modulate their speech production to overcome these transmission issues by, for instance, whispering, speaking louder or speaking clearer. In the literature, these modulations have been referred to as “speech modes”, “speech styles” or “speaking styles”. They are defined as specific variations of standard speech (SS), which refers to speech produced with normal vocal effort (Kelly & Hansen 2021; Tuomainen et al., 2022). Variations of SS (herein, speech modes) are used in daily conversations but have been surprisingly overlooked in the speech motor control literature. As a consequence, very little is known concerning the brain mechanisms that allow speakers to modulate their utterances. In this regard, one may wonder if speaking in a non-standard speech mode involves a motor speech preparation cost that reflects specific encoding processes. In the present study, we will exploit the high temporal resolution provided by the electroencephalography (EEG) brain imaging technique to investigate the encoding processes underlying the production of speech sequences under different modes. In the following sections, we will first unravel the issues underlying the scientific characterization of the motor speech encoding stage. We will then present the current knowledge about the targeted speech modes, loud and whispered speech, before trying to hypothesize how they may be encoded on the motor level.

### Motor Speech (phonetic) Encoding

Speakers can produce intelligible and accurate utterances almost automatically with a low error rate. Despite decades of investigation, a clear account of the interaction between the neural processes underlying speech production and their dynamics is still needed (Bohland et al. 2010; Laganaro 2019; Miller & Guenther 2021; Verwoert et al. 2022). Phonetic encoding [hereafter motor speech encoding processes] is the label given by some authors in the literature to describe the process of transforming an abstract linguistic sequence into a motor code readable by articulators (W. J. Levelt 1989; W. J. M. Levelt et al. 1999; Indefrey 2011; Guenther 2016). This encoding stage has been less studied in comparison to other language encoding processes resulting in a poor understanding of the underlying spatio-temporal dynamics (Indefrey 2011). The four level (FL) model from Van der Merwe (2020) proposed that motor speech encoding could be subdivided into two sequential substages; motor planning (i.e., retrieval of motor plans) and motor programming (i.e., where spatiotemporal and force dimensions are specified). This subdivision has been motivated by clinical symptoms of motor speech disorders such as apraxia of speech and dysarthria. To the best of our knowledge, the FL model is the only speech production model that provide some inputs regarding the encoding of speech modes. The model states that all the different speech modes can be grouped as a whole entity and would be thus encoded in the same way. Here, speech modes would be encoded through tuning of the unique suprasegmental features during the motor programming stage. Nevertheless, there is no empirical evidence corroborating this proposition. Studying the neural processes underlying speech modes as compared to standard speech can thus provide a relevant way of investigating whether speech modes require adjustments at specific encoding stages or whether they are supported by different encoding processes, as further presented below.

### Speech Modes

Speech modes constitute an omnipresent part of verbal exchanges. The way people speak is continuously influenced by intrinsic factors (speaker related) and extrinsic factors (environment or listener related) (Kelly & Hansen 2021; Smiljanić & Bradlow 2009; Whitfield et al. 2021). For instance, one needs to modulate his speech production to tell his friend the food he would like to order in a stadium full of supporters that are loudly singing an anthem. Moreover, meaningful cues (e.g., linguistic, affective or social cues) are conveyed to the interlocutor through the modulation of speech (Perkell 2012; Tourville & Guenther 2011). As an example, a speaker giving a talk during a conference will adopt a clear speech mode to emphasize his take home message. Zhang and Hansen (2007) proposed five speech modes with unique articulatory and phonatory features: whispered speech, soft speech, neutral (equivalent to standard) speech, loud speech and shouted speech. In this regard, it has been hypothesized that each speech mode involves its own mechanisms resulting in specific articulatory and phonatory patterns (Zhang & Hansen 2007; Scott, 2022). We will briefly describe the two speech modes that will be investigated in the present study, namely loud speech (LS) and whispered speech (WS).

### Loud Speech (LS)

When speakers struggle to convey a message to an interlocutor, for instance in a noisy environment, they usually modify their speech by increasing their vocal effort. In this case, increase in loudness leads to phonatory adjustments and changes in speech kinematics (Dromey & Ramig 1998; Huber & Chandrasekaran 2006; Whitfield et al. 2021). These manifestations associated to increased Sound Intensity Level (SIL) pertain to a specific speech mode labeled “LS”. Intuitively, one would conceptualize LS as the best speech mode to make yourself heard by someone else. However, the results obtained by Whitfield et al. (2021) indicate that LS’s main characteristic is increasing vocal intensity while there is not necessarily an improvement of the articulatory distinctiveness of the message conveyed. In the literature, LS has usually been associated to an increase in the standard SIL of 10 dB ± 4 dB (Huber & Chandrasekaran 2006; Whitfield et al. 2021). The encoding processes responsible for the increase in vocal loudness have not been clarified by functional neuroimaging or computational models, but some hypotheses on the proposed mechanisms will be presented below.

### Whispered Speech (WS)

WS is a widespread mode of communication aiming at conveying a message while remaining discreet. This speech mode is convenient in situations requiring silence (e.g., movie, theatre) or to keep private the content of a message (e.g., telling a secret). The ability to whisper is specific to humans (Tsunoda et al. 2011) and is characterized by reduced intelligibility and perceptibility for the listener as well as a more effortful production from the speaker’s point of view (Zhang et al. 2018). During whispered speech, physiological adjustments are applied to specific muscles of the larynx in order to prevent vocal folds vibration (Konnai et al. 2017; Solomon et al. 1989; Tsunoda et al. 2011). This absence of phonation provides unique features to WS. Actually, among speech modes, phonetic features of WS characterize it as the most distinct speech mode in comparison to SS (Kelly & Hansen 2021; Zhang et al. 2018; Zhang & Hansen 2007). Similar to LS, no consensus has been reached in the literature regarding the encoding processes responsible for whispering, leading to several proposed hypotheses.

### Encoding of Speech Modes

As anticipated previously, two possible hypotheses stem from the literature regarding the encoding processes associated to the production of loud and whispered utterances. On one hand, behavioral results (e.g., Huber & Chandrasekaran 2006) have led Whitfield et al., (2021) to hypothesize that an upregulation in the neuromotor drive is the mechanism at the origin of LS. However, it is unclear when and how this upregulation occurs in the motor speech encoding stage. On the other hand, two hypotheses were formulated based on functional Magnetic Resonance Imaging (fMRI) investigations in order to characterize the brain processes underlying WS. Correia et al. (2020) demonstrated that the fMRI response was greater for voiced speech than WS in the dorsal laryngeal motor area (dLMA), located in the primary motor cortex (M1). Under this hypothesis, the same brain mechanisms are at play for SS and WS, with larger recruitment for the former. A different hypothesis has been proposed by Tsunoda et al. (2011) based on a voluntary switching mechanism, in which ordinary speech would be transformed into whispering thanks to functional changes in the frontal lobe. However, their results showed two distinct patterns of brain activation in the frontal lobe involving both increased and decreased brain activation for WS relative to SS, which thus did not clarify how the functional switch would be carried out. In summary, the whispered speech’s literature gathers two distinct approaches concerning the encoding of WS: one described a functional difference in a specific motor region responsible for laryngeal control while another suggested the involvement of a voluntary functional switching mechanism during production of whispered utterances. In light of these theoretical propositions, speech modes could be encoded either (1) through neural adjustment of the same brain processes in the motor programming substage as proposed in the FL model or (2) through the involvement of an additional mechanism overlaying onto regular motor programming encoding processes. In particular, this study will explore these two hypotheses using behavioral and electrophysiological contrasts between speech modes and normally phonated speech. Specifically, LS (Experiment 1) and WS (Experiment 2) were compared to SS during a delayed production task of non-sense speech sequences (pseudowords). This paradigm is ideal to isolate motor speech encoding processes from linguistic encoding processes (Laganaro 2019, 2023; Piai et al. 2014). Electroencephalography (EEG)/Event-related potential (ERP) correlates of speech modes and SS will be analyzed during a time-window of about 350 ms preceding the vocal onset (hereafter referred to as “response-locked”) corresponding to the motor speech encoding stage. This time window is thus aligned to the vocal onset and analyzed in a backward fashion. In present study, we will exploit the high temporal resolution provided by the EEG to match the fast time scale of speech production processes (den Hollander et al. 2019; Laganaro & Perret 2011; Piai et al. 2014; Verwoert et al. 2022). Especially, we track the temporal dynamics of brain activations in the different experimental conditions via microstates analysis (Michel et al. 2009; Michel & Murray 2012; Murray et al. 2008) which will allow to investigate whether the encoding of speech modes elicit different brain processes relative to SS or if the same brain processes are engaged but with different dynamics.

## Experiment 1-Loud Speech

### Method

#### Population

30 French native speakers aged from 20 to 31 years old participated to the experiment. They were all right-handed [Average laterality quotient index = 88.33, range = 60–100] according to the Edinburgh Handedness Scales (Oldfield 1971). None of them had any neurological or motor impairment. Furthermore, participants had normal vision or corrected-to-normal vision. They all agreed to participate and signed the consent form accepted by the local ethics committee. They received a small financial compensation for their participation. 6 participants were removed due to either low production accuracy (i.e., below 75%), over-noisy EEG signal or being consider as an outlier in the Ragu Software (Koenig et al. 2011b). As a result, 24 participants (Mean (M) = 23.25 years old, Standard Deviation (SD) = 3.3 years, 5 males) were retained for the analyses.

### Material

The speech stimuli to be produced consisted of 67 monosyllabic and disyllabic pseudowords (see more details in Appendix A). Pseudowords were selected to avoid any linguistic effect related to words and thus focus on speech production. The pseudowords were composed of phonotactically legal French syllables according to the French database Lexique2 (New et al. 2004). All the items had the following syllabic structures: C_1_C_2_V_1_ – C_3_V_2_ for the disyllabic items (e.g., trafa) and C_1_C_2_V_1_ for the monosyllabic items (e.g., pra), with C_1_ being one of the three following voiceless plosives: /p/, /t/ or /k/.

### Procedure

The experiment took place in a soundproof room in which participants were seated at about 70 cm from the computer screen. The software E-Prime 3.0 (Psychology Software Tools, Pittsburgh, PA) was used to present the stimuli in several experimental blocks and to record participants’ productions.

**Fig. 1:**
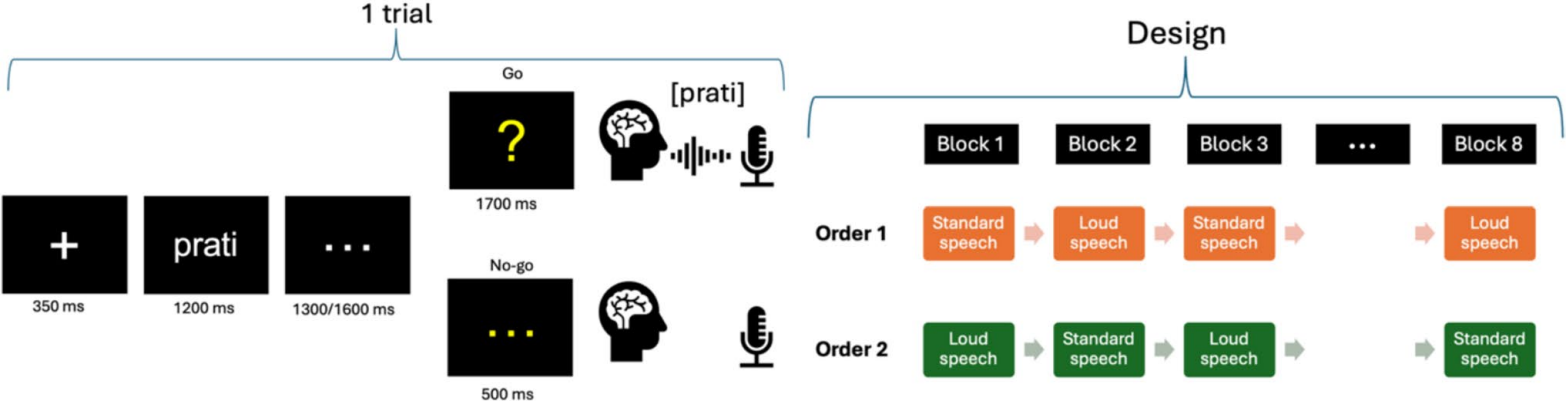
Illustration of the delayed production task on the left panel and of the experimental design on the right panel

Participants performed a delayed production task (see Fig. 1), in which they were asked to prepare a speech sequence based on a written pseudoword and to produce it aloud when a cue (here a question mark) appeared on the screen. Each trial displayed in succession a fixation cross (350 ms), a pseudoword written in white at the center of a black screen (1200 ms), a sequence of three dots indicating a variable waiting delay (either 1300 or 1600 ms) and eventually a yellow question mark appeared on the screen (1700 ms). The question mark was the cue indicating to the participants to produce the pseudoword previously presented as quickly and as accurately as possible. In some cases, yellow ellipsis dots appeared on the screen instead of the question mark indicating that no production were expected. These “no-go” trials, although not analyzed, were integrated to keep participants’ attention and to avoid anticipatory responses using a varying delay of either 1000 or 1900 ms. On average, no-go items appeared approximatively every nine trials.

Before the beginning of the experiment, participants read aloud a list containing all the stimuli to ensure they pronounced them correctly. Halfway through, they were asked to produce the rest of the pseudowords by adopting a loud speech mode. In cases of incorrect pronunciation, they were first corrected and then asked to produce the pseudoword in its correct form. A short training session with five pseudowords produced normally (three go and two no-go trials) preceded the experiment to ensure that the participants were comfortable with the experimental procedure. The experiment was segmented in eight experimental blocks, including four blocks of standard speech (SS) and four blocks of loud Speech (LS), presented in an alternated manner (see Fig. 1 right panel). Each block contained between 48 and 50 stimuli with both pseudowords and no-go items. Across the eight blocks, participants produced the same 180 pseudowords in each condition. Before each SS block, participants were asked to speak as usual. Before each LS block, participants were instructed to speak louder than usual, aiming at being heard from outside the soundproof room. To ensure that participants produced utterances that were loud enough during loud blocks, intensity was checked by the experimenters on a sound level meter which was hidden from the participants. Half of the participants started the experiment with a block in SS (order 1) and the other half with LS (order 2). Short self-paced breaks were given to the participants between blocks. Four lists including the 360 pseudowords were created and were randomly assigned to the participants to avoid order effects. The speech productions were recorded for off-line accuracy (ACC) check and extraction of vocal onsets (or reaction times, RT).

### Behavioral Analyses

Intensity was extracted and analyzed with the Praat software (Boersma & Van Heuven 2001). Speech intensity of all SS productions was averaged for each participant to establish an individual cut-off threshold. Therefore, loud utterances that were not higher than 8 dB in comparison to participant’s mean intensity were removed from the analyses. RT and ACC were extracted off-line through listening and visual inspection of the individual audio files with the Checkvocal Software (Protopapas 2007). Uncomplete (e.g., /kRat/ instead of /kRati/), uncertain (e.g.,/kr/…/kRata/) and incorrectly (e.g.,/kRotu/ instead of /kRutu/) produced pseudowords as well as productions that did not correspond to the target speech mode were considered as erroneous productions and were thus removed from the analyses. The vocal onset of each pseudoword was identified by aligning to the plosion bar produced by C_1_. Two judges (i.e., first and third authors) listened to the entire speech dataset resulting in an inter-judge agreement of 98% for ACC and 91% for RT. As cleaning procedure, RT with a SD above 2.5 of the mean latency of production per participant and per condition were removed. As ACC was not part of our hypotheses, this metric will be used as a descriptive statistic. The behavioral results on RT were analyzed using the Mixed Model approach (Bates et al. 2014; Carson & Beeson 2013) with the R-Software (R Core Team 2021). We compared multiple nested models that were built up by adding one effect at the time. The best model (see Appendix B) contained RT as dependent variable; speech mode (SS and LS), order of the experimental blocks (loud first or standard first) and length (monosyllabic or disyllabic) as fixed effects and subjects and items as random variables. Interaction effects between speech mode and order of experimental blocks were also tested in the model.

### EEG Recording and Preprocessing

The electrophysiological data was recorded continuously during the experiment with high density EEG using the Active-Two Biosemi EEG system (Biosemi V.O.F. Amsterdam, Netherlands) including 128 electrodes on the scalp with a sampling rate fixed at 512 Hz. All the preprocessing steps, including DC removal, filtering at 0.2 Hz (high pass) and 30 Hz (low pass), and Notch Filtering at 50 Hz to remove line current artifact, were done with the Cartool Software (Brunet et al. 2011). Each trial was inspected visually and excluded from the averaging if it was contaminated by any artifact (e.g., blinks, eye movements or noise). After visual inspection, epochs were extracted, matched in number across conditions and averaged per participant. Problematic electrodes were interpolated for each participant using 3-D splines interpolation (Perrin et al. 1987), with the same electrodes interpolated across the two uttering conditions. On average, 15.5 electrodes (range: 6–23) were interpolated per participant. Average reference was applied to the EEG data after interpolation. Eventually, we applied a spatial filter as a final step of the preprocessing procedure (see more details in Michel & Brunet 2019). Response locked epochs (i.e., aligned to the vocal onset) were extracted backwards with a time window of 175 TF (i.e., 342 ms). Epochs’ duration was selected based on the two reviews from Laganaro (2019, 2023). In the latter, it is suggested that motor speech encoding processes would take up to 300 ms of the planning time rather than the 145 ms proposed in the review of Indefrey (2011).

### Waveform Analysis

Electrodes’ amplitudes were compared between SS and LS with a massed approach on each electrode and time-point. This analysis was computed in the R software with the “threshold-free clusters-enhancement” (TFCE) method (Smith & Nichols 2009) using the permuco4brain R package (Frossard & Renaud 2021). This test has a high control over family-wise type I error. The analysis is based on 5000 permutation tests for repeated measure ANOVA.

### Topography Consistency Test (TCT)

The TCT (Koenig & Melie-García, 2010) aims at disentangling electrical sources from noise in the ERPs data with simple randomization techniques. In other words, this test tries to determine if the same brain topographies are obtained for a specific event with repeated measurements. Before computing topographical analyses, we applied L2 normalization of raw data to ensure investigation of qualitative differences. The TCT has been computed with the Ragu software (Koenig et al. 2011b) before performing the topographic and microstate (spatio-temporal segmentation) analyses. This step is fundamental because it constitutes a legitimate way of controlling that possible ERPs differences can be attributed to conditions and not to noise (Habermann et al. 2018).

### Topographic ANOVA (TANOVA) Analysis

By computing an index of dissimilarity, the TANOVA uses a non-parametric randomization test to determine at which time point ERP topographies (i.e. reference-free spatial distribution of the electric signal at scalp at a specific timepoint) significantly differ across conditions (Koenig et al. 2011a; Murray et al. 2008). The TANOVA analysis is complementary to spatio-temporal segmentation (see next analysis). Indeed, index of dissimilarity and the GFP exploits respectively the topographies and the response strength meaning that they can be measured and analyzed orthogonally (Murray et al. 2008b). A minimal duration threshold for significance can be calculated to control for the possible presence of false positives resulting from the dissimilarity analysis time point by time point (Koenig et al. 2011a).

### Microstates (Spatio-Temporal Segmentation) Analysis

The spatiotemporal segmentation of ERPs or microstates analysis is a two-step procedure aiming at representing conditions with several prototypical topographies or microstates maps corresponding to periods of quasi-stable topographies. Microstates analysis exploit the GFP value to decompose the signal into clusters of stable periods (60–120 ms) of electrophysiological activity (Koenig et al. 2014; Michel & Koenig 2018; Skrandies 1990). First, cluster maps were extracted from concatenated data across all conditions using the topographical atomize and agglomerate hierarchical clustering (T-AAHC) algorithm (see more in Murray et al., (2008)). These cluster maps are referred to as “template maps” and transition from one map to the other indicates changes in the global coordination of neuronal activity over time (Michel & Koenig 2018). The appropriate number of microstates maps were chosen on the basis of the cross-validation analysis provided by RAGU. During the clustering procedure, we applied a smoothing correction to get rid of sudden changes in microstates patterns due to noise. Here, 250 randomization tests of microstates models were computed to determine the best correlation between the grand mean explained variance and the number of microstates classes. Once cross-validation has been computed, the retained templates maps were fitted into participants’ individual signal for each condition in order to define the microstates and extract relevant parameters (Michel & Koenig 2018). These parameters represent several metrics of interest to describe EEG topographies: strength, timing and spatial distribution. In the present study, statistical analyses were carried out on one temporal parameter (duration, DUR) and one global measurement (area under curve, AUC) of occurrence. Note that no normalization or a-priori models have been applied during microstates analyses.

## Results

### Behavioral Results

The average intensity of production was 64.60 dB for loud utterances and 51.47 dB for standard utterances. The mean intensity difference was 12.73 dB (SD = 2.95 dB; minimum (Min) = 9.46 dB; maximum (Max) = 22.94 dB) on 148 loud trials on average. Participants produced pseudowords with a global high accuracy: LS utterances were produced with a 97% accuracy rate and SS utterances with 96%. The mean production latency for LS and SS was respectively 593.22 ms (SD = 118.41 ms) and 579.74 ms (SD = 124.28 ms). The best nested linear mixed model (see Appendix B for details) demonstrated a significant main effect of the speech mode (t(7365) = 4.85, β = 15.99, standard error (SE) = 3.295, p =  < 0.01), with LS yielding longer RT as compared to SS. Furthermore, a significant interaction effect was observed between the speech mode and the experimental block by which the participants started the experiment (t(7363.254) = -2.764, SE = 4.74, p = 0.005). Particularly, post-hoc analyses using a Tukey test showed that participants who started with a LS experimental block needed an additional initialization time of 16 ms to produce LS utterances (z = -4.852, SE = 3.3, p =  < *0.001*). On the contrary, the difference of estimate between LS and SS was not significant for participants who started with a SS experimental block (z = -0.851, SE = 3.4, p = *0.40*).

### ERP Results

#### TCT

Response-locked ERPs across conditions had an overall topographic consistency through the whole time periods (see Appendix D). Therefore, the whole resulting signal from the response-locked ERPs was kept for the following analyses.

### Waveform Analysis

**Fig. 2:**
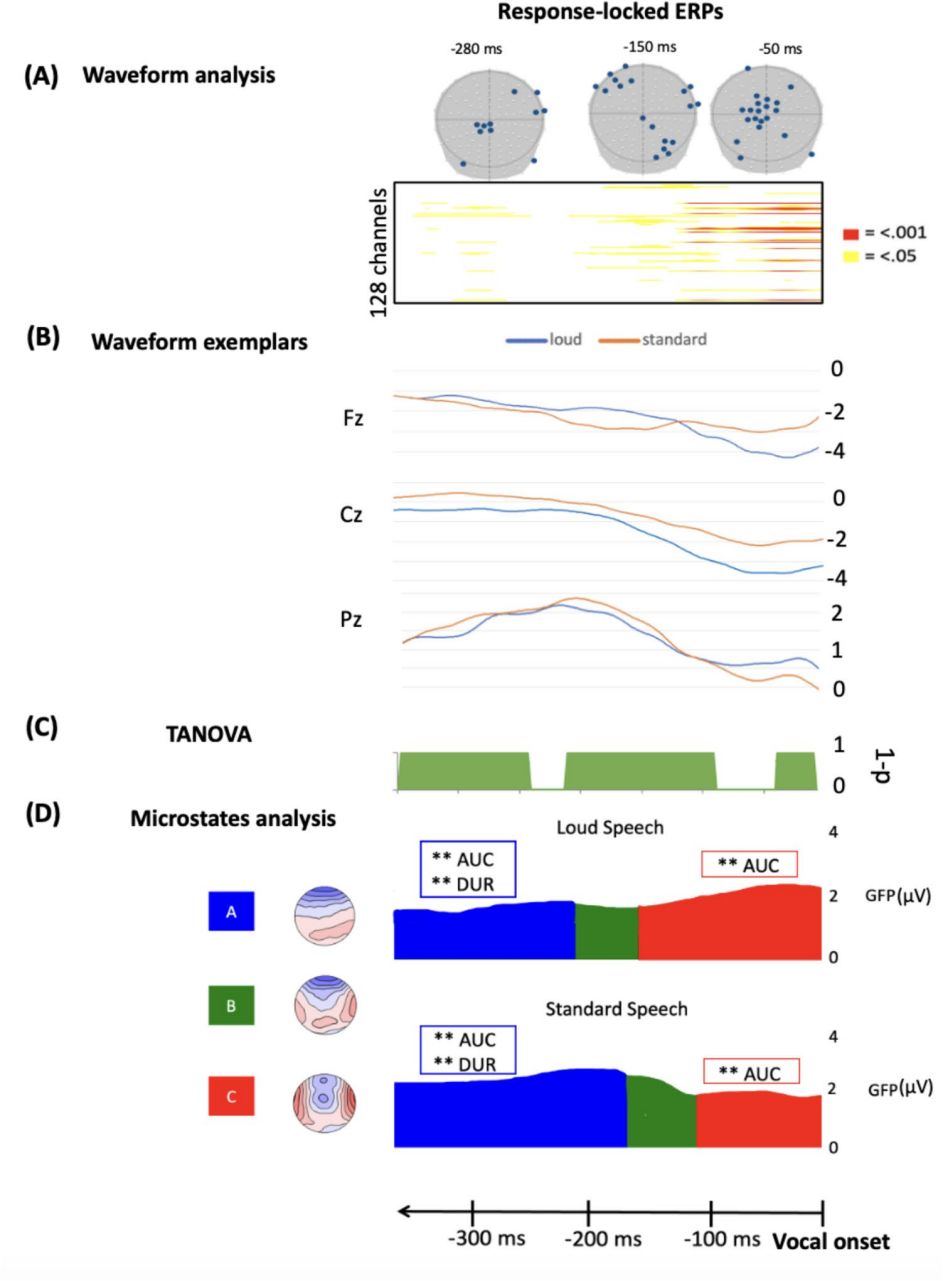
**A** Results from the waveform analyses across conditions on all time points (x axis) and electrodes (y axis) on response-locked ERPs, with red and yellow points indicating significant differences (p < .01 and p < .05, respectively). **B** Illustration of amplitude variations for the Fz, Cz and Pz electrodes. **C** Results of the TANOVA or topographic dissimilarity analysis with significant time periods represented in green (the y-axis represents 1-p values). **D** Results of the spatial–temporal segmentation across conditions represented on the mean GFP in microvolts (μV) per condition. The areas delimited correspond to each microstate with its associated topography (i.e., spatial distribution of the brain activity) on the left panel

The results of the test distribution of the TFCE procedure are presented on Fig. 2A. Amplitude differences in response-locked ERPs were observed at three time periods. From approximately -127 ms to the vocal onset (i.e., time 0), a cluster of 14 neighboring electrodes in the central-anterior region was found with lower values for the loud condition. Concerning the time period from -128 ms to-200 ms preceding the vocal onset, different amplitudes appeared on three small clusters: seven left anterior electrodes, four right anterior electrodes and five right central-parietal neighboring electrodes that tend to be more negative parietally and more positive anteriorly for the loud condition. Additionally, diverging amplitudes were observed in the -340 ms to -260 ms time period preceding the vocal onset on some sparse channels. Here, electrodes that significantly differed between the two uttering conditions had lower values for the loud condition.

#### TANOVA

Results of the pairwise TANOVA between LS and SS are presented in Fig. 2C. Green time periods correspond to significant time periods (< 0.05). Among the three significant periods presented, only one of them (i.e., from -225 ms to-100 ms) lasted longer than the duration threshold of 113.1 ms.

### Microstates Analysis

Three clusters were obtained for the response-locked spatio-temporal segmentation analysis, with 97.81% of explained variance (labelled from “A” to “C” in Fig. 2D). To statistically assess the global strength and duration of each map in the participants ERPs, the template maps were fitted in participants’ individual ERPs (see details on Appendix E).

Non-parametric Friedman test (Cleophas et al. 2016) demonstrated that the map A differed significantly across conditions on the two parameters of interest (AUC: X^2^(1) = 8.167, p = 0.004; DUR: X^2^(1) = 9.783, p = 0.004). In particular, map A lasted longer in the SS condition and had higher AUC value in comparison to the loud condition. On the other hand, the microstate map C significantly differed across conditions with the loud condition entailing a higher AUC value (X^2^(1) = 4.55, p = 0.033) compared to the standard condition.

## Discussion

In the present experiment, we investigated the behavioral and electrophysiological signature associated to producing an utterance when speaking normally or louder in a delayed production task. In the following, we will first discuss the longer RTs associated to the production of loud utterances before unravelling the electrophysiological correlates of this speech mode in response-locked ERP. The behavioral results replicate previous findings suggesting that producing loud utterances entails longer latencies in comparison to standard speech utterances (e.g., Zhang & Hansen 2007; Bourqui et al., submitted). Here, 16 additional milliseconds were needed for initializing loud utterances. This difference is inferior to the 34 ms differences reported in Bourqui et al. (submitted) but confirms that loud speech entails a behavioral encoding cost. However, this difference in RT depends on the mode by which participants started the experiment. This result will be discussed in the general discussion in the light of the second experiment.

On EEG/ERP signals, LS and SS differed in amplitudes, TANOVA and spatiotemporal segmentation in a large time window (i.e., from approximatively -220 ms to -100 ms before the vocal onset). This time-window falls within the time-window encompassing the last 300 ms preceding the vocal onset that has been associated to the motor speech encoding processes according to previous estimates (Laganaro 2019, 2023). Especially, producing loud utterances induces modulation of the waveform amplitudes during several time periods throughout the whole response-locked ERP, with larger amplitudes for LS in particular in the last 150 ms (see Fig. 2). Microstates are defined as periods with stable topographic representations suggesting quasi-simultaneity of activity among the brain regions involved in large-scale networks (Michel & Koenig 2018). Therefore, as the spatio-temporal segmentation and the fitting yielded the same sequence of microstates across conditions, we can claim that the production of loud and standard utterances is supported by the same brain networks and thus identical motor speech encoding processes. Although identical brain networks were found in both conditions, differences in temporal dynamics and strength of neural recruitment were revealed for maps A and C. In particular, the map A lasted longer and had a higher AUC value for the standard condition. In turn, map C demonstrated higher AUC value for the loud condition. In other words, these results suggest that to produce a louder utterance, the same neural processes as SS are recruited but with a difference in temporal dynamics and in the strength of recruitment. In particular, these changes in the temporal dynamics occur at two distant time periods corresponding to two different microstates (i.e., close and far from vocal onset) meaning that producing loud utterances involves probably more than just parametrization of muscle commands as proposed by Van der Merwe (2020). Before going further in the interpretation of the results obtained in this experiment, we will first investigate the behavioral and electrophysiological signatures of another speech mode with distinct phonatory and articulatory properties.

## Experiment 2-Whispered Speech

### Method

#### Participants

A different sample of 24 right-handed [Average laterality quotient index = 90.83, range = 60–100] neurotypical French speakers (M = 24.03 years, SD = 3.3 years, 10 men) fulfilling the same criteria as in Experiment 1 was recruited to perform the task.

### Material

The material was identical to experiment 1.

### Procedure

The experimental procedure was similar to experiment 1, except that this time WS was the speech mode contrasted with SS. During the training session, participants were instructed to speak without vibrating their vocals folds. For those who struggled to whisper, they did several extra items while focusing on not feeling vibration in their vocal apparatus. As in experiment 1, there were 8 blocks of approximatively 50 stimuli each, 4 performed in whispered mode and 4 in standard mode in a counterbalanced order across participants.

### Analysis

During the offline extraction of ACC and RT, WS’ intensity was increased to 70 dB to facilitate the alignment on the vocal onset using the Praat software. The inter-judgement agreement for the ACC and the RT was respectively 94% and 90%. On the behavioral level, the mixed model contained the same variables as the first experiment. On the EEG/ERP level, the length of the response-locked ERPs across conditions was 342 (i.e., 175 TF) as in the first experiment.

## Results

### Behavioral Results

High accuracy was obtained for both standard (M = 93.79%) and whispered utterances (M = 92.58%). Pseudowords in SS were produced on average latencies of 636.70 ms (SD = 167.95 ms) while whispered pseudowords required 652.91 ms (SD = 164.54 ms) to be initialized. The linear mixed model retained (see Appendix C for more details) showed a main effect of the stimuli’s length, with monosyllabic items yielding longer initialization time than disyllabic stimuli (F (7860) = 5.168, β = 6.410, *p* = 0.023), and a significant interaction effect between the speech mode and the experimental block by which the participant started the experiment. The post-hoc comparison with the Tukey test demonstrated that participants who started with a WS block had a significant 33.94 ms (z = -9.013, SE = 3.77, p =  < 0.001) longer initialization time in whispered utterances in comparison to standard utterances. On the contrary, no difference was observed across conditions in participant starting with a SS block (z = 0.367, SE = 3.74, p = 0.71).

### ERP Results

#### TCT

WS and SS response-locked ERPs did not contain any topographic inconsistency (see Appendix D).

### Waveform Analysis

**Fig. 3:**
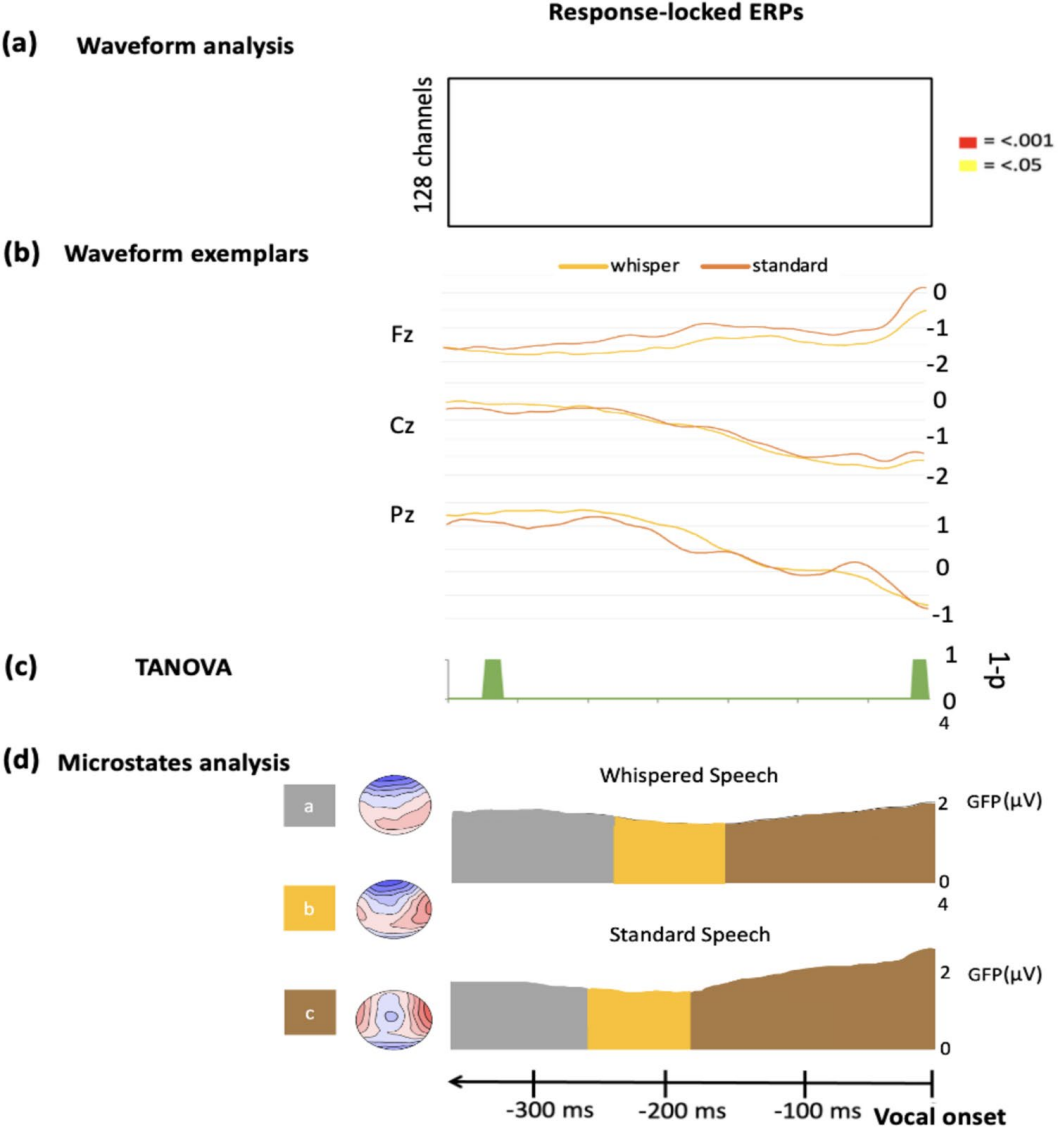
This figure illustrates the contrast between standard and whispered response-locked ERPs. **a** Results of the TFCE method revealing no significant differences in amplitude (p ≥ .05 for white) across conditions. **b** Fz, Cz and Pz exemplars of waveform modulations. **c** Results of the TANOVA analysis demonstrating short-timed differences in green between WS and SS. **d** Results of the spatio-temporal segmentation represented on the mean GFP in microvolts (μV) with the associated topographies on the left panel. Microstates or stable periods of electrophysiological activity are delimited by colors

The TFCE test for ERPs comparisons of amplitudes indicated no differences across conditions (see Fig. 3a) in the response-locked ERPs.

#### TANOVA

Response-locked TANOVA yielded two small time periods of significant difference across conditions (i.e., green time periods in Fig. 3c), which did not exceed the duration threshold of 52.65 ms.

### Microstates Analysis

The spatio-temporal segmentation of response-locked ERPs were segmented into three microstates maps explaining 97.34% of the global explained variance (GEV). Template maps were fitted to individuals ERPs to perform two statistical analyses. As in Experiment 1, we assessed the AUC and the DUR parameters (see details in Appendix F). The non-parametric Friedman test demonstrated that neither the AUC nor the DUR differed across conditions on any of the microstates maps.

## Discussion

In this second experiment, we contrasted the production of WS and SS utterances with the same pipeline as in the first experiment. In the present case, WS utterances required a longer production latency of 33.94 ms compared to SS. Although only observed on participants starting with a WS block, this result replicates previous findings (see Zhang and Hansen 2007; Bourqui et al., submitted) and differences across conditions are even larger than in those studies. As in experiment 1, the encoding cost observed in the behavioral results cannot be generalized as it depended on the first experimental block and will be further discussed in the general discussion. On the electrophysiological level, the same microstate sequences were observed in SS and WS, with only minor differences in the TANOVA analysis. However, these time windows did not exceed the significance duration threshold meaning that they could be the byproduct of false positives. The spatio-temporal segmentation and the fitting in the individuals further confirmed the absence of topographic differences across conditions. On the whole, it seems that electrical brain activity underlying the production of whispered utterances is not really different from producing standard utterances. The same finding has been reported previously on a smaller group of participants (Sikdar et al. 2017) suggesting that, on the electrophysiological level, whispering and speaking normally are similar in nature. Although coherent with a previous study, these results raise several questions that will be discussed in depth in the light of the first experiment.

## General Discussion

In this study, we investigated the behavioral and electrophysiological signatures of encoding the production of two distinct speech modes (i.e., loud speech (LS) and whispered speech (WS)) relative to standard speech (SS). Since the same procedure and material were used for both speech modes, we can broadly comment on the discrepancies and similarities across experiments. In the two experiments, behavioral results demonstrated longer initialization times for non-standard speech mode, with the result driven by participants that started the experiment with a block in the non-standard condition. This intriguing result may be interpreted as a “novelty bias” as speakers are not accustomed to speaking louder and whispering over such a long period of time. As a result, participants are potentially less familiar with the task and this behavioral encoding cost would thus need further investigation with another experimental plan. Current behavioral results however converge with previous studies using different paradigms and showing a cost of encoding non-standard speech modes (Zhang & Hansen 2007; Bourqui et al., submitted). Some authors have proposed that different speech modes with specific phonatory and articulatory features would involve unique encoding processes in comparison to standard speech (Scott, 2022; Zhang & Hansen 2007). Comparing the electrophysiological results across the present two experiments suggests that speech modes cannot be grouped as a whole entity encoded in the motor programming stage (i.e., last encoding process preceding articulation) as suggested in the FL model ( Van der Merwe, 2020). Indeed, EEG/ERP results do not converge as LS seems to entail important electrophysiological modulations while WS electrophysiological activity is very close to SS, a null result that has been reported previously (Sikdar et al. 2017). Particularly, LS electrophysiological activity differed in several times periods that seems to extend beyond the programming time-window, one close and one quite distant from the vocal onset. Our interpretation is that only the significant difference in strength of the last microstate preceding the vocal onset (map C in Fig. 2) could be considered as the “increase in neuromotor drive” proposed in the study of Whitfield (2021). The present results thus validate previous propositions by providing neuroimaging data indicating that speaking loud entails changes in temporal dynamics and an increase in brain activation during motor encoding. Additionally, they also replicate the finding from Sikdar et al., (2017) showing that WS and SS are similar on the electrophysiological level. In this particular case, the microstates results invalidate the idea that an additional mechanism is responsible for producing whispered utterances as proposed in Tsunoda et al., (2011). Indeed, the same microstates maps or the same encoding processes were found for both WS and SS. However, the dynamics of brain activation underlying these processes did not differ across conditions. In the light of the electrophysiological data, whispering cannot be distinguished from speaking normally and thus the literature should perhaps adopt a more nuanced approach to understand and characterize this mode.

Moreover, if the time-window of ERP modulations for LS seem to encompass a large portion of the time-window associated to motor speech encoding, likely planning and programming in the FL model, the present results can be also related to the neurocomputational framework of speech production and acquisition from Guenther (2016) named Direction Into the Velocities of Articulators (DIVA) model. Indeed, although there is no input so far on the dynamics of brain activation in the latter (Tourville & Guenther 2011), the present findings challenge our comprehension of the feedforward control system. If one assumes that the speech sound map (SSM, see more in Guenther & Vladusich 2012) corresponds to the motor planning in the FL model (i.e., where motor plans are retrieved) while the Articulatory map corresponds to the motor programming stage (i.e., where spatiotemporal and force dimensions are specified), our outcomes suggest that LS could be encoded somehow all along the process of activating the cells in the SSM and transmitting the motor targets to the Initiation Map and the Articulatory Map. For future studies, investigating speech modes thus seems to provide an interesting window to understand the intricate interplay between the functional units in the feedforward control system.

## Limitations

Some methodological concerns could be considered for future studies investigating speech modes and more especially for WS. On one hand, it has been suggested that there was an important intra-speaker and inter-speaker variability in the production of whispered utterances (Konnai et al. 2017). On the other hand, despite having the same instructions for every participant, we did not control for the type and/or the way participants were actually whispering. Effectively, Solomon et al. (1989) proposed that there are two types of whispering: quiet whisper (i.e., low effort manner) and a loud whisper (i.e., high effort manner) which were not controlled in this experiment. In brief, analysis of WS data implies several methodological challenges due to the inherent phonatory and articulatory properties of this mode of production.

## Conclusion

In the present study, we conducted two experiments which demonstrated that producing utterances under specific speech modes entails a behavioral encoding cost (i.e., increased production latency), although this result may need to be confirmed with a different experimental design. The EEG/ERP results demonstrated that speech modes with distinct phonatory and articulatory features cannot be grouped as a global entity and entail adjustments in different time-windows corresponding to different brain networks. Indeed, the electrophysiological signature of the two speech modes of interest were different with loud utterances entailing changes in ERP signal in two mental processes, one close and one further away from the vocal onset, while the ERP signal associated to whispered utterances did not differ significantly from standard ERP signal. These findings have important consequences as they challenge the current conceptualization of speech modes. Indeed, this study clarifies the statement “each speech mode possesses its own encoding mechanism” (Zhang & Hansen 2007; Scott 2022). Speech modes seem to be produced through the same brain networks as standard production but with a continuum of changes concerning the temporal dynamics and the strength of recruitment. These changes are observed in the whole motor speech (phonetic) encoding stage and they can range from important (in this case for loud utterances) to almost inexistent (in this instance for whispered utterances).

## Data Availability

Raw and pre-analyzed data supporting the findings of this study along with R scripts have been deposited on the institutional open repository Yareta(https://yareta.unige.ch/home); Doi:10.26037/yareta:gl46ca47tjgkridbttk2mnejke

## Appendix A

List of the 67 stimuli organized by syllabic structure and initial phoneme (p–t-k).

**Table:**

*Disyllabic*	prata	prouchi	ploussou	krapa	kroufou	klafi	trapa
	praka	proussi	plouti	krati	krouti	klata	trafa
	prassa	trafi	trachi	prati	krouchi	klati	tracha
	prafa	proutou	ploussi	krapi	kroussou	klacha	trapi
	pracha	proufou	ploufi	krassa	kroufi	klapa	plouki
	krafa	prouchou	plouchi	krafi	krouchou	klapi	proufi
	prafi	proukou	plouchou	krata	kroutou	klafa	ploutou
	prachi	proussou	ploukou	krassi	kroussi	klachi	prassi
	praki	prouti	ploufou	prouki			
*Monosyllabic*	pra	prou	plou	kra	krou	kla	tra

## Appendix B

*Table with the results of our best nested model: RT* ~ *(1 | subject)* + *(1 | stimuli)* + *speechmode* + *taskorder* + *syllabic structure* + *speechmode:taskorder.*

**Table:**

	*Estimate*	*Std. error*	*Degree of freedom*	*t-value*	*p-value*
*Standard speech*	*555.842*	*20.003*	*24.469*	*27.788*	< *0.001****
*Speech mode loud*	*15.988*	*3.295*	*7356.339*	*4.852*	< *0.001****
*Taskorder Standard First*	*55.243*	*28.214*	*24.210*	*1.568*	*0.13*
*Length monosyllabic*	*4.430*	*4.396*	*27.942*	*1.008*	*0.322*
*Loud * Standard First*	*− 13.092*	*4.737*	*7363.254*	*− 2.764*	*0.005***

## Appendix C

*Table with the results of our best nested model: RT* ~ *(1 | subject)* + *speechmode* + *taskorder* + *syllabic structure* + *speechmode:taskorder.*

**Table:**

	*Estimate*	*Std. error*	*Degree of freedom*	*t-value*	*p-value*
*Standard speech*	*661.674*	*34.718*	*24.165*	*19.058*	< *0.001****
*Speechmode whispered*	*− 1.375*	*3.744*	*7860.05*	*− 0.367*	*0.713*
*Taskorder Standard First*	*− 54.177*	*49.083*	*24.132*	*− 1.104*	*0.281*
*Length monosyllabic*	*6.410*	*2.820*	*7860.024*	*2.273*	*0.023**
*Whispered * Standard First*	*35.315*	*5.310*	*7860.036*	*6.651*	< *0.001****

## Appendix D

Results of the TCT for experiment 1 and experiment 2 with no period of topographic inconsistency across experiments.

## Appendix E

Results of the non-parametric Friedman test for response-locked microstates maps for experiment 1 on the Area under curve (AUC) and the duration (DUR).

**Table:**

	Parameters	Mean Standard	Mean Loud	X^2^	Degrees of freedom	Adjusted p-value (BH)
Map A	AUC	318.533	240.24	8.167	1	0.004*
	DUR	181.84	125.531	9.783	1	0.004*
Map B	AUC	56.273	70.258	0	1	1
	DUR	71.26	79.79	0.182	1	1
Map C	AUC	33.51	89.85	4.55	1	0.033*
	DUR	88.16	135.931	8.901	1	0.06

## Appendix F

Results of the non-parametric Friedman test for the response-locked microstates maps for experiment 2 on the area under curve (AUC) and the duration (DUR).

**Table:**

	Parameters	Mean standard	Mean whispered	X^2^	Degrees of freedom	Adjusted p-value (BH)
Map a	AUC	199.47	209	0.182	1	1
	DUR	147.8	143.731	0	1	1
Map b	AUC	51.71	51.125	0.73	1	0.79
	DUR	59.48	60.29	0	1	1
Map c	AUC	183.91	151.253	2.579	1	0.22
	DUR	133.981	137.231	0.53	1	0.47
